# Visualization and virtual screening in molecular property spaces

**DOI:** 10.1186/1758-2946-3-S1-P8

**Published:** 2011-04-19

**Authors:** A Klenner, M Reutlinger, G Schneider

**Affiliations:** 1 Institute of Pharmaceutical Sciences, ETH, Zurich, 8048, Switzerland

## 

**Figure 1:**
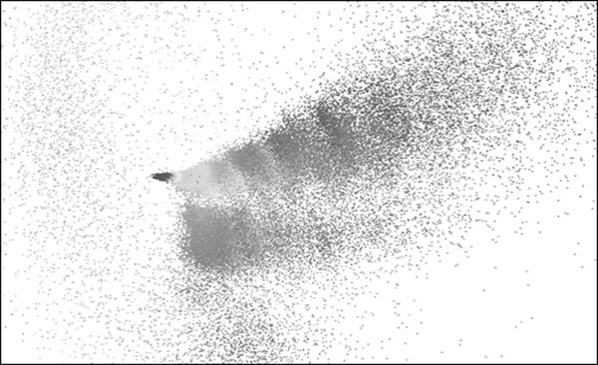
Projected property space of ~200.000 compounds from 90 dimensional space.

For virtual screening and similarity searching numerous descriptors can be employed to represent molecular structures and properties. Concurrently these descriptors always create a chemical property space. Typically, we lack information on how these spaces are structured and organized due to their high dimensionality. We present a projection method that allows for the visualization of such property spaces of large databases while maintaining the high-dimensional spatial structures and neighbourhood behaviour (Figure 1). The process of visualisation can help us understand how descriptors ‘perceive’ molecules and can give surprising insights which molecules are actually considered to be similar in these spaces. Furthermore we implemented a clustering algorithm using convex hulls for separating arbitrary molecule classes and automated feature extraction algorithm for identifying space defining features.

